# Our London Letter

**Published:** 1892-10

**Authors:** 


### Correspondence.

### LONDON LETTER.

Several disturbing factors have, during the past few months, been rampant, and have seemed to take away a portion of the interest in one's business, and now that politics and the weather are no longer the topics of conversation, the cholera epidemic is the subject which is being eagerly discussed. It has gained a great hold on the continent, and its effect on commerce is serious, particularly at Hamburg.

The Local Government Board of this country has issued voluminous instructions to the several local authorities, and the latter are making unstinted use of chloride of lime, carbolic acid, and other cheap and efficient disinfectants. Great attention is also being paid to the drainage system, and the general belief among medical men is, that the scourge cannot make much headway in England, although imported cases will naturally occur.

Sir Wm. Roberts, M. D., has been delivering some highly interesting lectures before the Royal College of Physicians here, and the matter explained, showed that a vast amount of reading and general research had been brought into requisition to place the lecturer in possession of the knowledge and acquaintanceship with the subjects dealt with.

As one result of his experiments regarding the treatment of gout, he comes to the conclusion that the use of alkalies, such as potash and soda are of minute, if of any real good, because the blood serum is already alkaline, and the addition of soda really hastens the precipitation of uric acid crystals. This, he remarks, is often the effect produced on a gouty patient by drinking alkaline waters.

The subject of cancer has been the subject of a series of lectures delivered by Dr. Britain at the Royal College of Surgeons. He dealt principally with his subject as found in chimney sweepers. It is curious that cancer is quite common among this class in Great Brittin, while in other parts of

Europe it is comparatively rare. Dr. Brittin accounts for this in various ways. In England, sweepers do not take much care as regards personal cleanliness, and they take no trouble whatever, to prevent the soot from reaching their bodies, or even their lungs, while abroad the sweeps exhibit considerable care, as a rule, to keep themselves protected from the soot. To attain this object, on many parts of the continent they wear a peculiar style of dress, generally tight-fitting and fastened round the wrists and ankles by tapes or straps, to leave as little space as possible for the ingress of soot. They also cover their mouths and noses with a cloth, and all these precautions are taken regularly, as a matter of course.

The soot most likely to produce a cancerous condition, is that of the coal known as hard or stone coal.

At the meeting of the British Medical Association at Nottingham, several highly interesting and instructive addresses were given, and among these, Dr. Brookhouse, the senior physician to the Nottingham General Hospital, gave some notes on his treatment of phthisis by menthol. Since the practical abandonment of Dr. Koch's treatment, intra-trachial injection has come more to the front, various substances being used, such as eucalyptus and creasote in castor oil, or aristol in the same medium, but Dr. Brookhouse recommends the simple menthol solution; he injects once or twice daily into the trachea of his patients, one drachm of a twelve per cent. solution of pure menthol in pure olive oil, using a laryngoscopic mirror and an ordinary tracheal syringe. In his cases where this treatment was used, he says that very soon there was marked decrease both of cough and expectoration, the temperature was much steadier and there was a gain in weight, night sweats being lessened or altogether checked. Although, as a palliative measure, menthol may be of great use as a curative agent, the results have not been very satisfactory.

The British Association Conference held in Edinburg was a great success and was very largely attended by members from all parts of the world. The work gone through was very great, and embraced a large selection of subjects. Dr. Louis Robinson gave an interesting lecture on the prehensile power of new-born infants. He remarked that [a new-born child would hold on to his hand and support its weight in the air for a minute and sometimes even thirty seconds longer. He traced this back to a time when it was necessary for the young one to hold on to its parent, and so leave the parent's hands free, as seen in the arboreal apes at present.

The much boasted cure for cancer, perfected by Count Mattei, has come to a hasty termination, now that the report of the committee appointed by the Matteists to watch cases of their selection has been issued. The cure, as many eminent and reputable practitioners supposed, is clearly entitled to be regarded as a swindle, and it is a pity that attempts to dupe those suffering from painful maladies cannot be prosecuted. The defense would probably be that all was done in the interests of science and suffering humanity in general!

